# Ketogenic Diet: Parental Experiences and Expectations

**DOI:** 10.1177/08830738241227066

**Published:** 2024-02-05

**Authors:** Elizabeth Orr, Robyn Whitney, Nandini Nandeesha, Eric H. Kossoff, Rajesh RamachandranNair

**Affiliations:** 1 Department of Nursing, Faculty of Applied Health Sciences, 7497 Brock University, St. Catharines, Canada; 2 Division of Neurology, Department of Paediatrics, 3710 McMaster University, Hamilton, Canada; 3 School of Nursing, 3710 McMaster University, Hamilton, Canada; 4 Departments of Neurology and Pediatrics, Johns Hopkins University School of Medicine, Baltimore, MD, USA

**Keywords:** ketogenic diet, pediatric epilepsy, qualitative research, quality of life

## Abstract

**Background:**

The ketogenic diet may be difficult for some patients and their families to implement and can impact physical, emotional, and social well-being.

**Methods:**

Through principles of fundamental qualitative description, we completed an exploratory study on parents’ experiences and expectations on the use and efficacy of the ketogenic diet for children with medically refractory epilepsy.

**Results:**

Seventeen parents (10 mothers and 7 fathers) of 12 children with epilepsy participated. At the time of the interview, parents had experienced an average of 25 months of ketogenic diet treatment for their child (range 2 months to 98 months). Half of the caregivers learned about the ketogenic diet from their neurologist, whereas the remainder had heard about it from another source (ie, the internet). Most caregivers’ (n = 13) diet expectations were related to seizure control. However, child development (n = 5) and quality of life (n = 5) were also crucial to some. Physical impacts of the diet were most commonly gastrointestinal for children (n = 9). Social and emotional effects were noted in some older children with typical development. Most caregivers described negative impacts on finances (n = 15), relationships (n = 14), and emotional well-being (ie, stress) (n = 12). Caregivers benefited from the ketogenic diet team's regular communication, close follow-up, and family-centered care.

**Conclusions:**

Despite the impacts that the ketogenic diet may have on caregivers’ emotional and social well-being, the positive impacts of the diet were felt to outweigh any perceived risks. Effects (both positive and negative) on quality of life and child development (eg, social, emotional, cognitive) are essential for caregivers and require additional investigation.

Ketogenic diet therapy is an accepted therapy for children whose epilepsy is refractory to antiseizure medication.^1^ The ketogenic diet has been used to treat children with intractable epilepsy since 1921, when the original high-fat, low-carbohydrate therapy was created. The limited intake of carbohydrates triggers the body to enter a state of ketosis by using fats as its primary fuel source. Being in a ketotic state is associated with decreased seizure frequency, although the full extent of its mechanisms is not well understood.^2–5^ About 50% of children with drug-resistant epilepsy treated with a ketogenic diet experience a greater than 50% reduction in seizure frequency.^1^ The diet is highly beneficial in epilepsy populations(ie, >70% than the average 50% seizure reduction), especially those with Dravet syndrome, tuberous sclerosis complex, infantile spasms, glucose transporter 1 deficiency syndrome, and epilepsy with myoclonic atonic seizures.^2,6–9^

The ketogenic diet can be burdensome to families in both time and financial resources. Families referred to the ketogenic diet program often ask if the diet will work for their child, but factors delineating responders from nonresponders within epilepsy types are variable.^10–14^ Various types of ketogenic diet have been designed to improve palatability or reduce adverse side effects. A range of products, resources, and family support groups are available to help people following ketogenic diet therapy. The ketogenic diet works quickly when effective, typically within the first 1-2 weeks,^15^ but we usually ask families to try it for 3 months before discontinuation.

However, adherence to a dietary regime may be challenging. Nonadherence to treatment may be unintentional, for example, due to forgetfulness, limited resources, or intentional, where people consciously decide not to follow an agreed treatment plan. Nonadherence is multifaceted and depends on physician-related and health care system-related factors, but ultimately, the patient chooses whether to adhere to treatment. A critical element in understanding intentional nonadherence to treatment is the individual's beliefs about treatment—specifically, their perceived personal need for treatment and concerns or worries about treatment.^14,16–18^

There have been studies to examine parental goals and expectations before starting ketogenic diet therapy.^19^ Seizure control is important, but even more crucial may be cognitive improvement. Recognizing these goals is essential to be realistic with families before embarking on ketogenic diet therapy. However, knowing these goals can help at follow-up visits to judge true efficacy (and therefore continue the ketogenic diet if appropriate or discontinue if not). Therefore, we aimed to better understand parents’ expectations and experiences with ketogenic diet therapy in children with medically refractory epilepsy using in-depth, exploratory one-on-one interviews.

## Methods

### Study Design

The principles of fundamental qualitative description were used to guide this exploratory qualitative study of parents’ experiences and expectations on the use and efficacy of the ketogenic diet as a therapeutic option for medically refractory epilepsy. Qualitative description is a form of naturalistic inquiry that allows a rich, straight description of an experience or event in language that is similar to the informants’ own language (ie, less abstract), making it particularly amendable to practitioners’ questions and policymakers.^20,21^

### Participants

A purposeful sample of parents of children who received (July 1, 2014–June 30, 2017) or were on classic ketogenic diet to treat epilepsy at the time of the interview was recruited from the “Diet Therapy for Epilepsy” database at McMaster Children's Hospital, Hamilton, Ontario, Canada. Patients who were initiated on an urgent basis for status epilepticus were excluded. All children were started on the diet for seizure control as the indication.^2^ All patients in the cohort were initiated on the ketogenic diet with precise prescriptions of daily macronutrients and calories as part of the diet plan.^22^ A Neurology nurse who was part of the clinical care team contacted the potential parent participants to inform them about the study and obtain verbal consent to be contacted by the research assistant. The research assistant then conducted the initial screening of participants, discussed the project in detail, and obtained consent to participate in a one-on-one telephone interview.

### Sample Size

Sampling in qualitative research is an iterative process, so the sample size is not determined a priori. Based on the nature of the topic, the scope of the study, and our previous research experience designing qualitative research in the field,^23,24^ a sample size of 15 participants was estimated to reach informational power.

### Data Collection

Data were collected by a research nurse using in-depth, one-on-one telephone interviews (Table 1) with both mothers and fathers. Interviews lasted between 30 and 75 minutes. A semistructured interview guide was used to explore the experiences and expectations of parents when using the ketogenic diet as therapy for their child's epilepsy; topics included parent expectations before initiating ketogenic diet; the impact of ketogenic diet on their child's life, family life, and other relationships; impacts on work and finances; as well as perceived outcomes of the ketogenic diet in their child. Furthermore, the interview also addressed interactions with the ketogenic diet team and support those parents required with diet implementation. All interviews were digitally recorded and transcribed verbatim.

**Table 1.** Semi-Structured Interview Guide.

(1) Please take a minute to remember when you were told your child had epilepsy. Can you describe this experience to me? (a) When you were hearing this information for the first time, what did you find helpful? (b) During this period, what did you not find was supportive or helpful? (c) Looking back, what would have been the ideal circumstances under which to learn that you had epilepsy? (2) When you first learned about epilepsy, what were your main concerns or worries? (3) Explain how you heard about the ketogenic diet for the first time. (4) Would you have preferred to have been informed about the ketogenic diet differently? Please explain. (5) Please explain why you started your child on KD, and your expectations from this form of treatment initially. (6) Please explain your initial concerns about the KD therapy before initiation. (7) How would you describe success in KD therapy? (8) How did the implementation of the ketogenic diet affect your child? (9) How did the implementation of the ketogenic diet affect you? ( (10) Could you explain your (and your child's) experience with the ketogenic diet therapy team and their recommendations? (11) From your experience, what were the benefits and drawbacks of ketogenic diet in the treatment of epilepsy? (12) In your opinion, once the physician discusses the option of ketogenic diet therapy with a patient, what additional follow up measures should the physician and the team take to support the patient and family? (13) What hospital support systems would you suggest once the patient is initiated on diet? (14) In your opinion, what is the role of community epilepsy agencies in supporting patients on ketogenic diet?

Abbreviation: KD, ketogenic diet.

### Analysis

Interview transcripts were stored and managed using the qualitative software NVivo11 (QSR International Pty Ltd 2018). Inductive content analysis was used to organize, code, and categorize the interview data.^25^ First, transcripts were read and reread to make sense of the data as a whole. Next, key concepts from the interview guide and emerging concepts from the open coding process were grouped into categories. Categories were then further grouped to form broader categories that provide a means for describing the experience of interest. Two researchers participated in the data analysis to ensure the dependability of the coding and categorization of data. Each researcher independently coded the transcripts; then codes and categories were compared and discussed until agreement was reached.

## Results

### Parental Expectations and Experiences of Ketogenic Diet

Thirty-two parents were contacted (16 mothers and 16 fathers). Twenty-one parents consented. However, 4 could not participate in the interview after the consent process. A purposeful sample of 17 parents (10 mothers and 7 fathers) participated in the study; 5 were couples. Twelve children with epilepsy were represented in this sample. At the time of the interview, parents had experienced an average of 25 months (range: 2-98 months) of ketogenic diet to treat their child's epilepsy (see Table 2 for participant demographics). The results were organized by content analysis and described in categories and supporting subcategories (see Table 3).

**Table 2.** Demographics of Participants.

CWE^a^	Parent	Parent age (y)	Age of CWE at the time of interview (y)	Diagnosis	KD ratio and seizure control before DC or interview	Length of time on KD before DC (mo); reason for DC	When the KD was discontinued before the interview (mo)
1	M	41	7	Metabolic, congenital hyperammonemia hyperinsulinemia syndrome	1.59:1; >50%	7^b^	NA
2	M	41	9	EMAS, presumed genetic	1.5:1, Sz free	64; Rx completed	15
3	M	39	12	EE, genetic, KCNT1 mutation	1.8:1; <50%	2; frequent infection, poor tolerance and Sz control	23
	F	40					
4	M	37	7	EMAS, presumed genetic	2.1:1; Sx free	43; Rx completed	3
	F	37					
5	M	46	13	LGS, presumed genetic	3:1, >50%	98^b^	NA
6	M	33	1	EE-IS, presumed genetic	3.5:1, >50%	10 ^b^	NA
7	M	30	2	Structural, polymicrogyria	2.5:1, <50% ^c^	4 ^b^	NA
	F	29					
8	M	61	18	Presumed genetic	1.3:1, <50% ^c^	13 ^b^	NA
9	M	32	1	EE-IS, presumed genetic	4:1, <50%	6 ^b^	NA
10	M	33	1	EE-genetic SCN8A-related epilepsy	4:1, <50%	3; No Sz control	0
	F	33					
11	F	32	3	EE-IS, presumed genetic	4:1, <50%	19; No Sz control	9
12	F	61	7	Genetic, GLUT-1 deficiency	2:1, Sz free	9 ^b^	NA

Abbreviation: CWE, child with epilepsy; DC, discontinuation; EE, epileptic encephalopathy; IS, infantile spasms; EMAS, epilepsy with myoclonic-atonic seizures; F, father; KD, ketogenic diet; LGS, Lennox Gastaut Syndrome; M, mother; NA, not applicable; Rx, treatment; Sz, seizure.

^a^ Cases 2, 4, 8, 10: Oral feeding, rest enteral tube feeding.

^b^ Length of time from diet initiation to the interview (for patients still on the diet at the time of interview).

^c^ Parental preference to continue KD.

**Table 3.** Results Categories and Subcategories.

Learning about their child's epilepsy
Learning about the KD
Decisions to start the KD
Expectations prior to the KD
Concerns prior to the KD
KD success defined
Impact of KD on the Child
Physical and emotional/mental impact
Social impact
KD and the parents/caregivers
Physical and emotional/mental impact
Social impact
Financial and work impact
Effects of the KD
Benefits and drawbacks of the KD
Experience with the KD team
Desired follow-up and support
Role of community epilepsy agencies and KD

Abbreviation: KD, ketogenic diet.

## Learning About Their Child's Epilepsy

Before the discussion of ketogenic diet, parents were asked to describe the experience of learning their child had epilepsy. Parents described several intense emotions, including horror, shock, devastation, terror, grief, helplessness, fear, and confusion. Descriptions of several negative experiences, including misdiagnosis, perceived delay in diagnosis, and difficulty accessing specialized care, accompanied the emotional responses described.

Actually it was a very long procedure because no one actually thought he had epilepsy. I explained it to multiple doctors and I kind of explained it like a tick cause that's how long it lasts . . . a couple of seconds and it was finally the endocrinologist that said that, that looks like an absence seizure, so he finally set me up to see a neurologist.

At the time of diagnosis parents were primarily concerned about the health and well-being of their child (eg, death, developmental delay, quality of life). They also valued being taken seriously by care providers, access to the appropriate level of care/expertise, a clear plan of care, and both informational and emotional support.

## Learning About the Ketogenic Diet

Parents described first learning about ketogenic diet from several different sources, with just over half hearing about the diet from the neurology team (n = 9) and a half from other sources (n = 8); “I think it was something that I’d read about on the internet. Just trying to bring other possible treatment options to the doctors.” Most parents (n = 10) described ketogenic diet being introduced as an option by the neurology team following the failure of other treatment options, primarily medications; “I think I’d read about it previously that it was used sometimes to treat with diet, but it really only became consideration for her when we were told that she was intractable, her condition was intractable.” The timing of ketogenic diet being discussed varied in length of time from diagnosis of epilepsy but always followed the failure of antiseizure medications.

## Decisions to Start the Ketogenic Diet

Parents described the decision to start the ketogenic diet as a “last resort” or because they felt they were running out of options (n = 11); “and we had nothing left to try, there was nothing we could do, so it was either accept it for what it is, or try the ketogenic diet”; “well she was on five medications, and we were running out of options.” Despite starting the diet as a perceived last resort, for some parents, it offered renewed hope in the treatment of their child's epilepsy (n = 6); as one parent described,

Once we knew a little bit more about how promising the diet was for [child's] condition, then it kind of gave a new hope because every time we’re starting a new medication and we’d have more seizures happening, it was really disheartening to be trying more medications because it felt like we were just repeating the same pattern over and over again.

### Expectations Prior to the Ketogenic Diet

Parent expectations of the ketogenic diet were primarily related to seizure management (n = 13); however, child development (n = 5) and quality of life (n = 5) were also described. The expectation of seizure control from the ketogenic diet was described as ranging from the complete absence of seizures—“I think in the beginning it was to get-100%-seizure-control, and our goal was to get her off of all the pharmaceuticals”—to a reduction in frequency or magnitude: “We were quite okay with some seizures, even daily seizures we were okay with, we just wanted to see him have less.” Parents expected that with seizure control would come improved quality of life for the child and a better chance at child development (eg, social, emotional, cognitive). As one parent described, “We thought that with the seizure control, the child would actually start to develop a little better, maybe start talking … motor skills and stuff like that.” For parents where epilepsy was a secondary or associated diagnosis, expectations and concerns were often described within this context.

### Concerns Prior to the Ketogenic Diet

Parental concerns before initiating the ketogenic diet included time, preparation, and planning (ie, the perceived amount of effort the change in diet would entail) (n = 10); social and family implications (eg, travelling while on the diet, family mealtimes, and associating food with socializing, pleasure, and/or reward) (n = 7); health of the child (eg, side effects and nutrition) (n = 4); and cost (n = 6). When describing concerns related to preparation and planning, the feeding method was often depicted with a distinct contrast between oral and tube feeding plans, with tube feeds being preferred to oral. For example, this parent of a gastrostomy tube–fed child described in their reaction to implementing the diet, “If we had to make meals and go out with those meals and worry about oral foods, I think my answer would be a bit different.”

Some parents expressed little concern before initiating the diet (n = 3), for example, “there weren’t really any concerns. I thought strictly it was beneficial,” or they felt they knew so little about the diet that they were unsure what to be concerned about (n = 2); “My biggest fear had nothing to do with difficulty following it per se because I didn’t really fully understand it yet, I just knew that there was a lot of things that they couldn’t eat.” Although parents expressed concerns with ketogenic diet, many considered it worthwhile compared to seizing as one parent described: “But because I was seeing for 24 hours a day how much she was seizing, those things didn’t even really cross my mind because all I could think was how bad her quality of life was when she was constantly seizing. Nothing that the diet could compare to—if it had efficacy—nothing could compare to how bad her quality of life was when she was seizing. So I’d rather have her at a party eating some different foods rather not at the party at all because she was too sick to be there.”

### Ketogenic Diet Success Defined

When asked, it was difficult for parents to reflect on their definition of ketogenic diet success before starting the diet (all parents interviewed had already initiated the diet with their children). Instead, many described how successful the diet was for their child rather than this ideal result. However, for those who did define ketogenic diet success, seizure management was the ideal outcome; as this father described, “The main benefit would obviously be seizure-control; I don’t know any other benefits to it because we wouldn’t do it for fun.”

## Impact of Ketogenic Diet on the Child

Parents described ketogenic diet's physical, emotional/mental, and social impact on their child. How the diet was delivered (oral vs enteral feeds) and the child's developmental status often influenced the diet's perceived impact on the child. The child's age at the time of diet initiation also appeared to affect the perceived impact of the diet on the child, especially the emotional/mental and social impact of the diet on older children.

### Physical and Emotional/Mental Impact

Some families reported adverse effects, including gastrointestinal issues (vomiting, constipation, ileus, etc) (n = 9). Two families, however, reported improved levels of consciousness (n = 2), weight gain (n = 2), and effects on sleep (4 described no effect on sleep or improved sleep, and 2 parents said a negative impact on sleep). Several parents reported no physical impact of ketogenic diet on their children (n = 6).

Most parents described little emotional problems related to eating the ketogenic diet with their children. This was often attributed to the child's young age or developmental status; for example, this parent described her young child's response to the diet, “Honestly, he was so good. I can’t image implementing the ketogenic diet on a child who's 6, 7, 8, or 9 . . . that would be far more difficult than it is for a 2-year-old. So he took to it actually very well, tolerated eating the same sort of meals over and over and over pretty well.” In contrast, some families (n = 5) reported difficulties. One parent of an older child described all pleasure being removed from eating and that food was “literally like taking medication for her.”

### Social Impact

Similar to emotional/mental impact, parents who described a negative effect of the ketogenic diet on their child were generally parents of older, typically developing children (n = 3). Birthday parties, holidays, and time with friends were when the diet impacted the child socially. The parent of the oldest child in the sample described a profound social impact: “I would say especially for a 17-18-year-old girl, [the diet] has really limited her a lot in terms of her ability to blend in and to be a part of high school life.”

## Ketogenic Diet and the Parents/Caregivers

Despite describing some of the more challenging aspects of ketogenic diet, an overarching theme was that as a parent, you “do what you have to do” for the health and well-being of your child even despite potential physical, emotional/mental, social, and financial impact for the parents.

### Physical Impact and Emotional/Mental Impact

Parents described very little effect of being on the ketogenic diet on their physical health. Any physical impact seemed to be more generally about having less time or energy to care for themselves because of their child's unwellness or the diet's time demands.

Most parents spoke about the impact of ketogenic diet on their emotional or mental health and well-being (n = 12). Stress was the primary issue described; however, in some cases, this stress was not attributed directly to the ketogenic diet but to their child's overall health. As this parent described, “and now we’re realizing that this is most likely going to be a life-long journey and that we’re not doing as good as thought, like we’re not managing the stress as well.”

### Social Impact

The social aspect of ketogenic diet seemed to have the most significant potential difficulty reported by parents compared with the other areas discussed. Some discussed being able to integrate ketogenic diet into many of their usual routines and activities—“We just kind of adapted to it and tried not to let it affect our lives as much as possible,” whereas others found it very restricting or incompatible with social activities—“we stopped doing anything. We stopped socializing, we stopped having people over, we just stayed home.” Two areas of social life that seemed mainly involved by the ketogenic diet were family dynamics and relationships (with the child and others). Parents often described changing family dynamics or taking on different or more defined roles than before ketogenic diet. Additionally, although parents typically described feeling supported by their partner, one parent often took the lead with the ketogenic diet, resulting in most of the work and responsibility falling on themselves. Most parents described the diet as impacting their relationships with others (n = 14). Some parents described the diet as bringing them closer together with their partner parent, whereas others expressed strain on this relationship. One parent found constantly explaining the ketogenic diet to friends and family tiresome. At the same time, another felt the pressure on the relationship with their child, describing feeling like their “personal chef.” A theme throughout the parent experiences of ketogenic diet was difficulty trusting others with administering the diet for their child (eg, teachers, educational assistants, babysitters, extended family, and even partner parents); this led some parents to feel isolated and worried about what would happen to their child if something happened to them.

### Financial and Work Impact

Most parents (n = 15) described some impact on finances and work. Parents who worked outside of the home described having to take time off work to manage their child's illness and the demands of the diet. Parents also described the financial impact of the diet, with some having to modify their previous family lifestyle to accommodate the accumulating costs of the diet. As this parent described, “everything is exceedingly expensive, like all those types of special products, and there's no funding.” Most parents described being fortunate enough to have the resources to afford the costs associated with the diet but were concerned with how “it's just it's adding up.”

## Effects of the Ketogenic Diet

Results of the ketogenic diet as described by parents ranged from successful (n = 6; eg, “seizure-free,” “improved management,” “life changing”) to neutral (n = 4; eg, “successful and not successful at the same time”) to unsuccessful (n = 5; eg, “I don’t think I would call it a success”). Although parents could not explain why the ketogenic diet worked from a physiologic perspective, for those who experienced success, this was attributed to strict adherence to the diet protocol—“there was no ‘yes you can have a bite of a cracker,’” synergy between the diet and medications, and access to the ketogenic diet team and appropriate resources.

### Benefits and Drawbacks of the Ketogenic Diet

The “main benefit” of the diet described by parents was seizure control (n = 7). Other benefits of the diet included improved quality of life (n = 3), better child cognitive development (n = 2), and decreased use of or improved response to medications (n = 2). Parents described several drawbacks of ketogenic diet; however, if there were benefits, these would “outweigh” the disadvantages. Drawbacks included time, organization, and planning; side effects; cost; and food restrictions or an altered relationship with food (eg, “It was hard to figure out a way to reward without being food-based”).

## Experience With the Ketogenic Diet Team

Overall, parents’ experiences with the ketogenic diet team were positive. Most parents described effective communication with the team (n = 14), noting the ability to ask questions by phone or email and receive prompt answers. When speaking of their experiences with the ketogenic diet team, parents also described aspects of child- and family-centered care (n = 7), including personalized care and collaboration. As this parent described, “they’ve all been great, and they all take our opinions and our recommendation with [child]. They listen to us, which is a big thing.” Close monitoring and follow-up (n = 4), support (n = 5), and expertise (n = 2) were also described as positive aspects of working with the ketogenic diet team. One parent whose child was aging out of pediatric care was concerned about the transition to adult care, highlighting this as a specific area for improved communication and follow-up.

### Desired Follow-up and Support

When asked what additional measures or follow-up the ketogenic diet team could take when supporting families with ketogenic diet therapy, responses closely mirrored the descriptions of experiences with the ketogenic diet team. Parents desired consistent and timely communication; they wanted someone to listen to their concerns and help solve problems when they were encountered—this was described as having phone or email access to a member of the ketogenic diet team—not just scheduled visits. It was also important for parents that this communication continued beyond the initiation phase of the diet. Parents also described social support as an additional measure for supporting ketogenic diet families. This included access to information or additional resources (including informal resources or experiential knowledge, eg, tips and tricks), emotional support (eg, parent support group, Facebook page), and access to instrumental support (eg, financial aid).

## Role of Community Epilepsy Agencies and Ketogenic Diet

Many parents were unaware of or could not describe the role of the community epilepsy agencies in supporting ketogenic diet parents (n = 7). Those parents who did describe a role for the community epilepsy agencies often discussed their potential role in connecting parents to resources, including forms of support (informational, emotional, instrumental). Creating awareness of ketogenic diet within the epilepsy community and a broad understanding among the general population was also seen as a primary agency function (this was especially important for parents considering recent “keto” lifestyle and diet trends).

## Discussion

Our study examined several factors related to ketogenic diet therapy and its positive and negative impacts on the emotional, physical, and social well-being of children with epilepsy and their caregivers. Most caregivers viewed ketogenic diet therapy as a last resort for seizure control and viewed seizure control as the main benefit. However, impacts on quality of life and cognitive development were also noted. For children on the ketogenic diet, physical impacts most often occur in the form of gastrointestinal complaints, which are common with ketogenic diet therapy. Emotional impacts were seldom reported in our cohort. However, for children with normal cognition, the ketogenic diet was found to have impacts on social functioning as well. Although physical impacts were not observed in caregivers, emotional and social impacts were. Caregivers reported increased stress, effects on relationships, trust issues (ie, when it came to others administering the diet), and financial constraints because of ketogenic diet therapy. Although caregivers felt supported by their ketogenic diet team, they were primarily unaware of how community agencies could help them through ketogenic diet therapy.

Parental expectations and experiences about ketogenic diet therapy can be understood only in the context of diagnosing a chronic illness like epilepsy. Overall, negative emotions and experiences reported by the participants in this study may be related to the diagnosis of epilepsy in their children and not to ketogenic diet therapy itself. Similar reactions have been reported in prior studies.^26–31^ Caregivers often fear nocturnal seizures,^28^ the unexpectedness of seizures,^30^ the potential implications of seizures on their child's brain,^31^ and death or serious injury.^26^ Several caregivers expressed feelings of worry, shock, upset, and anxiety following the diagnosis of epilepsy in their child in our study, which is similar to previous reports.^26^

Although more than half of the participants had learned about ketogenic diet from the neurology team, it was often introduced only after failure with multiple antiseizure medications. A survey of child neurologists in the United States in 2001 confirmed that 82% used ketogenic diet only after “most new antiseizure medications” had been attempted and failed. However, 89% of child neurologists reported having previously used the ketogenic diet.^12^ An anonymous survey among parents of 107 consecutive children initiated on the ketogenic diet reported that the most common source of information about ketogenic diet was the physician who referred the family for the ketogenic diet, with 77 (72%) responding that this played a role in their decision. The Internet was the next most frequently reported, with 60 (56%) acknowledging that they had obtained information from the Internet or an Internet-based support group.^32^ Our study showed similar results, with half of the participants reporting they had heard about ketogenic diet therapy from other sources, such as the Internet, which could result in misinformation.

Updated recommendations of the International Ketogenic Diet Study Group concluded that ketogenic diet should be strongly considered in a child who has failed 2 antiseizure medications.^2^ Thus, it is important to emphasize to the families that ketogenic diet therapy should not be viewed as a last resort but as an effective form of therapy for children with epilepsy. Family support websites like the Charlie Foundation and Matthew's Friends highlight this.

Pre–ketogenic diet, parental expectations were mainly related to seizure management (ie, seizure freedom and reduction, stopping or reducing antiseizure medications) in our study. However, the development of the child and improved quality of life were also part of parental expectations. These expectations were coupled with concerns about the time commitment, meal preparation, and planning; implications on family dynamics; child health; and cost of ketogenic diet therapy. Many parents described the success of ketogenic diet in terms of seizure management. Based on the evaluation of 100 consecutive pre–ketogenic diet letters from parents expressing their goals of ketogenic diet therapy, Farasat et al reported that the most common first goal for families was seizure improvement, the second was antiseizure medication reduction, and the third was cognitive improvement. Most caregivers (90%) in this study requested improved cognition or alertness.^19^ Prediet evaluation and counseling are important to establish goals of care and expectations concerning ketogenic diet therapy.^2^

Our study revealed that ketogenic diet therapy affects aspects of the child's life, that is, physical, emotional, and social domains. The age of the child influenced how they were impacted. Negative physical impacts were related to gastrointestinal system side effects (ie, constipation). These effects are not uncommon during ketogenic diet therapy, especially during the initiation period.^3,33^ A few caregivers reported positive benefits in weight gain, improved level of alertness, and improved or little to no change in sleep quality. In a cohort of 14 children, Ulalp et al reported improved sleep quality in 7 of 14 patients (50%), deterioration in 5 patients (35.7%), and no change was seen in 2 on ketogenic diet therapy (14.3%).^34^ For children with normal cognition, there were some negative social and emotional impacts of ketogenic diet therapy reported by caregivers (ie, impacted social outings, birthday parties, etc). Strategies to help in these social situations are important to guide families starting the ketogenic diet. Because of the effects that strict ketogenic diet therapy can have, it is generally recommended that adolescents with normal cognition should be offered the option of more liberal and flexible forms of ketogenic diet.^35^

Emotional, mental, social, and financial impacts were described in parents’ lives, not only their children. It is unclear whether the reported difficulties were due to ketogenic diet, the child's illness, or both. Ketogenic diet therapy could challenge parental identity given that negative meanings tend to be associated with high-fat foods and children's food consumption is seen as the parent's responsibility. In-depth interviews with 12 parents by Webster and Gabe showed that although the meanings attached to foods are ingrained and difficult to challenge, they are not necessarily fixed.^16^ Parents could reverse the negative connotations attached to fat by viewing food as medicine. The authors also reported that the meanings attached to food might be more likely to be altered if dietary treatment is successful when previous treatments have had limited efficacy.^16^ Caregivers in our study reported increased stress, effects on relationships, trust issues (ie, when it came to others administering the diet), and financial constraints because of ketogenic diet therapy. However, despite these findings, it was agreed that the benefits of ketogenic diet therapy outweighed any negative aspects. Our results are similar to a recent caregiver survey, which found that despite challenges, the majority felt supported and happy with ketogenic diet therapy for their child and observed improved quality of life.^10^ Unfortunately, patterns suggesting why the diet was easier or more difficult to integrate did not emerge in this study but could be an avenue to explore in the future.

Quality of life (QoL) is an important outcome measure for children with chronic health conditions and their families, including epilepsy. There has been growing interest in the quality of life of patients with refractory epilepsy treated with the ketogenic diet to improve compliance, because poor adherence to the treatment could worsen clinical conditions.^18^ A few authors have studied the validity of QoL instruments in this regard.^11,13,36^ An interdisciplinary team that uses a comprehensive approach to implementing the ketogenic diet may result in increased family satisfaction and improved dietary compliance, which maximizes the diet to its fullest potential and thus may, in turn, impact QoL. These outcomes are validated by anecdotal information from families’ satisfaction reports and successful diet maintenance for increased months.^10^ In addition, child life support involvement has been proven useful, especially for parents during the initiation week, and may be worth utilizing to relieve stress.^37^ Close follow-up, support from the ketogenic diet team, and family-centered care were important to several caregivers in our cohort. Many caregivers were not aware of the role of epilepsy agencies in ketogenic diet therapy, and strengthening this relationship would be important. We recommend a comprehensive upfront discussion with caregivers about the practical expectations and the variables that could be assessed following the implementation of the ketogenic diet as important components for the successful partnership between the caregivers and the ketogenic diet team. A recent publication on the core outcome set of childhood epilepsy treated with ketogenic diet therapy mentioned that seizure reduction/freedom, antiseizure medication use, adverse effects of ketogenic diet, dietary compliance, tolerability of ketogenic diet, behavioral feeding difficulties, “parents feel supported to manage ketogenic diet,” assessment of parent's confidence with the provision of ketogenic diet, and quality of life for a child on ketogenic diet as important outcomes.^38^ Both parents and health care providers participated in this study.

Overall, the main strength of our study is our unique study design. Limitations of the study included a small cohort, single-center study, and we had only children on a single form of dietary therapy (where the daily calories and macronutrients were precisely prescribed). Therefore, it is to be determined whether the results of our study are generalizable to all comers on other forms of ketogenic diet therapy. Although, importantly, our cohort did contain a range of participants, such as responders (ie, >50% seizure control) and those with no seizure control. Because of the limited sample size, we did not do a separate analysis for parents of responders and nonresponders, as well as based on the duration of ketogenic diet therapy. These data also did not include parent opinions before starting the ketogenic diet, and even theoretically afterward, which would help clarify parental perceptions of the ketogenic diet over time. It is quite possible that the participants in our current study were “information seekers.” Hence, the opinions expressed by the participants in the study may not be representative of all the parents of children treated with ketogenic diet therapy for epilepsy. This study is also susceptible to recall and recruitment bias, as parents who dropped out of ketogenic diet were not included. Furthermore, given that the study interview was conducted by an individual not previously known to the family, it is possible that parents did not feel comfortable discussing the emotional impacts of the ketogenic diet over the phone. These impacts may have been underestimated as a result. Moreover, given that 5 couples were included in this study, it may have also biased the results of this study. Additionally, it is unclear whether the results of this study are transferable to all geographic, cultural, and ethnic backgrounds, as we did not formally document the ethnicity or cultural backgrounds of the participants. In addition, our results may not be transferrable to other forms of ketogenic diet therapy (ie, low glycemic index therapy, modified Atkins diet, etc). Although the sample size was limited, informational power was enhanced by the emergence of the same themes across different personal interviews. The sequential development of a survey instrument built on the findings from this qualitative research will be necessary.

## Conclusion

Our study highlights that although effective, ketogenic diet therapy can positively and negatively impact emotional and social well-being, especially for children with normal cognition. For caregivers, impacts were observed on emotional and social well-being. Despite the challenges with ketogenic diet therapy, caregivers believed the benefits outweighed any perceived risks. Seizure control is viewed as the main benefit of ketogenic diet therapy, although impacts on quality of life and cognitive development are also important. Close follow-up, support from the ketogenic diet team, and family-centered care are important for families.
